# Elastic Resistance and Shoulder Movement Patterns: An Analysis of Reaching Tasks Based on Proprioception

**DOI:** 10.3390/bioengineering11010001

**Published:** 2023-12-19

**Authors:** Gyuseok Shim, Duwon Yang, Woorim Cho, Jihyeon Kim, Hyangshin Ryu, Woong Choi, Jaehyo Kim

**Affiliations:** 1 Department of Human Ecology & Technology, BrainKorea21 FOUR, Handong Global University, Pohang 37554, Republic of Korea; gyuseoksa@handong.ac.kr (G.S.); doonebest0730@gmail.com (D.Y.); 2 Department of Information and Communications Engineering, Tokyo Institute of Technology, Yokohama 226-8503, Japan; cho.w.ab@m.titech.ac.jp; 3 Department of Digital Healthcare, Human Integrated Solution, Goyang 10464, Republic of Korea; jihyeon@hisolution.co.kr; 4 College of ICT Construction & Welfare Convergence, Kangnam University, Yongin 16979, Republic of Korea

**Keywords:** elastic resistance, shoulder press, proprioception, reaching, OpenPose, skewness, exercise intensity, symmetry

## Abstract

This study departs from the conventional research on horizontal plane reach movements by examining human motor control strategies in vertical plane elastic load reach movements conducted without visual feedback. Here, participants performed shoulder presses with elastic resistances at low, moderate, and high intensities without access to visual information about their hand position, relying exclusively on proprioceptive feedback and synchronizing their movements with a metronome set at a 3 s interval. The results revealed consistent performance symmetry across different intensities in terms of the reach speed (*p* = 0.254–0.736), return speed (*p* = 0.205–0.882), and movement distance (*p* = 0.480–0.919). This discovery underscores the human capacity to uphold bilateral symmetry in movement execution when relying solely on proprioception. Furthermore, this study observed an asymmetric velocity profile where the reach duration remained consistent irrespective of the load (1.15 s), whereas the return duration increased with higher loads (1.39 s–1.45 s). These findings suggest that, in the absence of visual feedback, the asymmetric velocity profile does not result from the execution of the action but rather represents a deliberate deceleration post-reach aimed at achieving the target position as generated by the brain’s internal model. These findings hold significant implications for interpreting rehabilitation approaches under settings devoid of visual feedback.

## 1. Introduction

In everyday life, humans execute numerous reaching movements, employing a combination of the brain’s internal model [1], visual information [2], and the intricate interplay of the musculoskeletal system [3] to achieve precise targeting. Notably, among all body joints, the shoulder joint offers the highest degree of freedom for these movements [4]. The control of the shoulder joint during reaching movements is primarily influenced by two key factors: muscle fatigue [5] and visual feedback [6]. Muscle fatigue can significantly diminish the force-generating capacity of the shoulder’s surrounding muscles, posing a challenge to maintaining movement performance. Within this context, visual feedback assumes a pivotal role. It refers to the sensory information received through the eyes, which aids in coordinating and adjusting movements based on visual cues [7]. However, when visual feedback is unavailable, reliance shifts to proprioception. Proprioception, or the proprioceptive sense, recognizes body position and the necessary force adjustments without relying on visual inputs [8,9]. While proprioception is crucial for movement control, it inherently lacks the precision offered by visual feedback [10]. When visual feedback is unavailable and elastic resistance is introduced as an external force, an essential component of proprioception, known as movement feedback, experiences enhancement [11]. This enhancement results in an increased level of accuracy in reaching movements. Elastic bands provide a resistance that intensifies as the distance from the neutral position increases, thereby furnishing finely tuned feedback for joint position and muscle force control [12]. This directly contributes to the enhancement of proprioception.

Past studies have predominantly examined the influence of load on reaching movements within a two-dimensional horizontal plane with visual feedback [13,14]. Human movements typically follow straight trajectories from the initial point to the target, characterized by symmetric, bell-shaped velocity profiles [15], as elucidated by the minimum variance model [16] and the minimum torque-change model [17]. When an elastic load is applied, the minimum variance model predicts asymmetrical velocity profiles due to the influence of the elastic force, whereas the minimum torque-change model anticipates symmetrical velocity profiles regardless of the magnitude of the external force. Nevertheless, human movements encompass not only horizontal but also vertical components. Thus, for a comprehensive understanding of human reaching movement control, it becomes imperative to analyze reaching movements across various planes, considering the presence or absence of visual feedback.

The primary objective of this study was to examine the strategies governing movement control during reaching motions within a vertical plane, particularly when visual feedback was absent and elastic resistance was introduced. In this context, participants engaged in shoulder presses utilizing elastic bands, emphasizing the compensatory role played by somatosensory feedback and the impact of muscle fatigue when experiencing elastic resistance. Generally, when individuals rely solely on somatosensory feedback, an expected outcome is an asymmetry in the movement speed between the dominant and non-dominant arms, attributable to differences in sensory reliance [18]. Furthermore, exercises that primarily employ somatosensory feedback are anticipated to prioritize the minimization of muscle force fluctuations over the reaching accuracy, likely resulting in symmetrical velocity profiles [19]. This approach can advance our comprehension of proprioception development in diverse reaching movement contexts and offers a more intuitive framework for movement analysis.

## 2. Materials and Methods

### 2.1. Subjects

Thirty healthy participants, with an average age of 22.8 ± 1.97 years and no physical impairments, were selected for this experiment. All participants were provided with comprehensive written and verbal explanations detailing the experiment’s purpose, background, precautions, and procedures. Subsequently, written informed consent was obtained from each participant. This study received approval from the Institutional Review Board of Handong Global University (Approval No. 2022-HGUA020) on 25 September 2023. Table 1 provides a summary of the participants’ information. The participants were recruited through the use of advertisements. During the initial screening process, questionnaires were employed to evaluate all participants for the presence of any physical injuries. Only those individuals without injuries were considered eligible for participation in the experiment. The inclusion criterion for participation in the study was either no prior experience in resistance exercises or less than two months of experience. Exclusion criteria included a maximum muscle strength lower than 2.63 kgf for each arm and the presence of physical or mental disabilities.

### 2.2. Experimental Setup

In this experiment, participants performed a band shoulder press. The bands used were TheraBand elastic bands (TheraBand, Performance Health, Akron, OH, USA) with seven different resistance levels ranging from the yellow-colored band with minimum tension (1.36 kgf, 100% of elongation) to the gold-colored band with maximum tension (6.44 kgf, 100% of elongation). As the elastic bands stretched, the resistance increased. The maximum joint range of motion (ROM) of the participants was measured to ensure a consistent maximum band tension in the experiment. Half of the measured length from the arm to the body was set as the length of the elastic band. This helped ensure that the elastic load did not exceed 100% during exercise.

To analyze the participants’ movements, we captured videos using an RGB camera (STREAMCAM, Logitech, Lausanne, Switzerland). The camera supported a resolution of 1080 p and recorded 60 frames per second (fps). The LogiCapture software 2.08.12 (Logitech, Lausanne, Switzerland) was used to record videos at 60 fps. The camera was positioned on a tripod at a height of 1 m from the ground and a distance of 2.2 m away from the participants in the frontal view.

**Figure 1:**
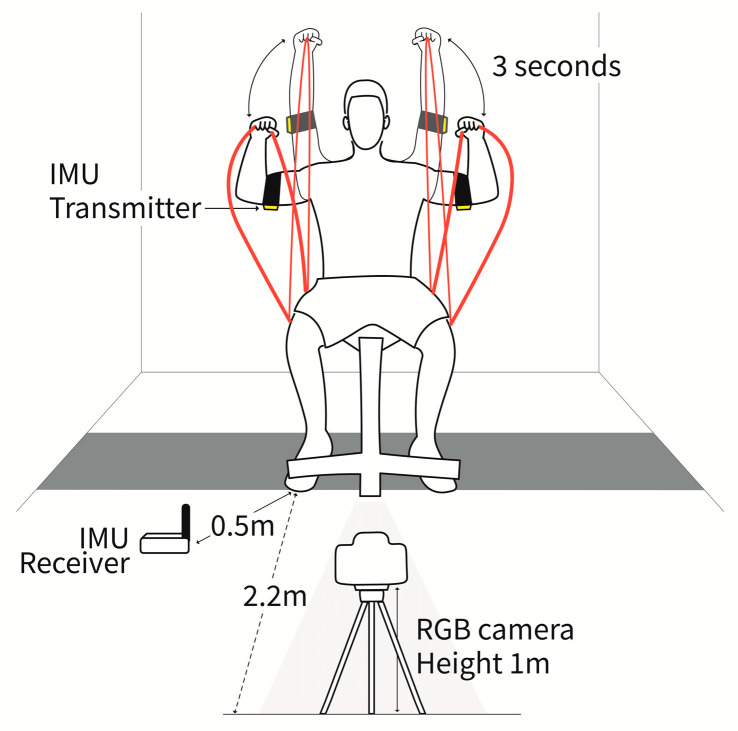
Schematic of the experimental environment. The RGB camera was positioned 2.2 m from the participants and at a height of 1.1 m from the ground. Each participant repeatedly performed lifting and lowering actions in sync with a metronome that rang at 3 s intervals.

To validate the accuracy of the joint positions estimated by OpenPose (version 1.6.0) from the camera data, inertial measurement units (IMUs) were attached to both forearms in front of the elbows of the participants. The IMUs consisted of a transmitter (EBIMU24GV5, E2BOX, Hanam-si, Gyeonggi-do, Republic of Korea) and a receiver (EBIMU24GV5, E2BOX, Hanam-si, Gyeonggi-do, Republic of Korea) capable of recording quaternion, gyro, and acceleration data. The IMUs were sampled at 60 fps to match the camera data. The receiver was attached to the ground and placed approximately 50 cm in front of the participants to ensure that it did not interfere with their movements (Figure 1).

To minimize the influence of psychological factors and individual variations in exercise repetition speed, a metronome with a tempo set at 20 beats per minute was employed [20]. Per the metronome’s auditory cues, participants raised and lowered the elastic band in synchrony with each sound. The metronome function was programmed using MATLAB R2021b and transmitted through the speakers of the measurement equipment, a Precision 7760 laptop (Dell Technologies Inc, Round Rock, TX, USA).

### 2.3. Experimental Procedure

#### 2.3.1. 1–Repetition Maximum Measurement Session

Initially, a session was conducted to measure the 1–repetition maximum (1 RM) to set the weights according to different exercise intensities. First, an explanation of the shoulder press posture was provided to the participants. A familiarization session followed this to ensure accurate performance in terms of the movements. Female participants performed the exercises without employing an elastic band, whereas male participants utilized a yellow band, and each group completed the exercise regimen five times. This methodological choice was based on the hypothesis that the lower maximum muscular strength typically observed in females might impede their ability to attain accurate posture when guided by the lightest resistance band. By contrast, considering that the maximum muscular strength in males is estimated to surpass the weight of the lightest band by more than five-fold, using a yellow band was deemed suitable for this group. Subsequently, starting with the yellow band, the participants incrementally performed a single shoulder press for each stage. The posture was considered correct when the elbow level exceeded the height of the nose when the arms were fully extended. A successful lift was recorded if the participant performed the shoulder press within a sufficient range of motion; otherwise, it was considered a failure. Upon successfully lifting the weight, the participants rested for 2 min before attempting to lift the next weight in the following order: yellow, red, blue, green, black, gray, and then gold. The measurement was repeated after successful attempts with the gold band, starting with the lighter bands. The weight in the previous successful attempt was considered 1 RM if the participant could no longer perform a single repetition or failed to achieve the standard range of motion.

#### 2.3.2. Intensity-Specific Exercise Sessions

After a 3-day rest period, intensity-specific exercise sessions (low, high, and moderate) were conducted. The intensity criteria were as follows:

- Low intensity: bands with a weight less than or equal to 40% of 1 RM [21].
- Moderate intensity: bands with a weight greater than or equal to 50% of 1 RM but less than high-intensity bands.
- High intensity: bands with a weight greater than or equal to 80% of 1 RM [22].

The experiment was conducted in the following order: low, high, and moderate intensity. Eston et al. reported, using the Borg-15 scale, that individuals perceived weight as “low” in intensity when it corresponded to 40% of their 1 RM (one-repetition maximum). Furthermore, Morishita, S. documented that a weight perceived as “high” in intensity aligned with over 80% of the 1 RM. Because a paucity of research explicitly defines the percentage of 1 RM associated with the “moderate” intensity level, an intermediate weight falling between the “low” and “high” categories was established for this categorization. This was repeated for a maximum of 15 times [22] or until the participant could no longer lift.

If the elbow did not touch the height of chin or the movement did not match the beats per minute, the number of repetitions was recorded, and the participant was asked to rest for a certain period. After each session, a 5 min rest period was allowed, during which participants assessed their exercise intensity using the Borg 15-point RPE scale. After 5 min, the participants were asked about muscle fatigue. If fatigue was experienced, an additional 2 min rest period was allowed to minimize the impact of muscle fatigue.

#### 2.3.3. Openpose Validation Session

Eight randomly selected participants were chosen to validate the OpenPose data following the intensity-specific exercise sessions. The selected subjects wore IMU sensors in front of their elbows and performed 15 repetitions with a yellow band.

### 2.4. Data Analysis

#### 2.4.1. Data Processing

The recorded videos were processed using the OpenPose to estimate the joint positions. The exercise data of the participants were represented as individual positional data using the Body_25 format. Upon conversion of the data to the Body_25 format, matrices representing pixel data $x_{i},y_{i}$ for joints in the human body were obtained. The vertical positions of the endpoints of the right and left hands were denoted as $y_{4}\;and\;y_{7}$, respectively.

A first-order low-pass filter with a cutoff frequency of 0.5 Hz was applied to remove outliers and high-frequency noise from the data of the participants. The transformed data were recorded in terms of pixels. Notably, individual body lengths or situational factors can influence the analysis of the data in pixel coordinates. Therefore, the z-score technique was employed to normalize the OpenPose data for each participant. Each normalized joint position was obtained using the following:

(1) $$x_{i}=\frac{x_{i}-\mu }{\sigma }$$

Here, $x_{i}$ refers to the joint position data, while μ and σ denote the mean and standard deviation of the joint position data during one trial, respectively.

#### 2.4.2. Openpose and IMU Comparison

To validate the precision of the OpenPose technology, a comparison was conducted between the shoulder angle measurements obtained from both the IMU and OpenPose. The OpenPose approach involved the computation of the shoulder angle utilizing vector mathematics.

$$vector_{1}=\left(p_{2x}-p_{1x},p_{2y}-p_{1y}\right)\;vector_{2}=\left(p_{3x}-p_{1x},p_{3y}-p_{1y}\right)\;\theta =\cos^{-1}\left(\frac{vector_{1}\cdot vector_{2}}{\left|vector_{1}\right|\cdot \left|vector_{2}\right|}\right)\;p_{1x},p_{1y}=x_{2\;or\;5},y_{2\;or\;5}/p_{2x},p_{2y}=x_{9\;or\;12},y_{9\;or\;12}/p_{3x},p_{3y}=x_{3\;or\;6},y_{3\;or\;6}$$

This process facilitated accurate estimation of joint positions and the computation of joint angles by utilizing OpenPose’s advanced pose estimation algorithms. By contrast, the IMUs yielded a distinct yet complementary angle estimation. The quaternions recorded by the IMUs were meticulously converted into Euler angles, representing roll, pitch, and yaw. When the IMUs were placed on the participants during the band shoulder press exercise, the roll angle corresponded to the shoulder angle in the frontal plane. This specific angle was then compared to the angle derived from OpenPose.

- RMSE (Root Mean Square Error): To understand the error between the two measurement techniques, the root mean square error method was employed. Here, $y_{PP}$ represents the estimated angle of the shoulder from Openpose, and $y_{IMU}$ is the angle estimated from the IMU. n denotes the length of data for each measurement.
  $$RMSE=\sqrt{\frac{1}{n}\sum_{i=1}^{n}{\left(y_{PP}-y_{IMU}\right)}^{2}}$$
- Correlation coefficient (CC): To analyze the correlation, the CC for the angle of the shoulder measured using both the IMU and OpenPose was calculated. A CC value of 0.8 or above indicated a strong correlation between the two measurement methods. The coefficient was calculated using the following formula:

(2) $$CC=\frac{\sum_{i=1}^{n}\left(y_{PP}-{\bar{y}}_{PP}\right)\left(y_{IMU}-{\bar{y}}_{IMU}\right)}{\sqrt{\sum_{i=1}^{n}{\left(y_{PP}-{\bar{y}}_{PP}\right)}^{2}}\sqrt{\sum_{i=1}^{n}{\left(y_{IMU}-{\bar{y}}_{IMU}\right)}^{2}}}.$$

Here, $y_{PP}$ is the angle of the shoulder obtained from OpenPose, $y_{IMU}$ is the angle obtained from the IMU, and ${\bar{y}}_{PP}$ and ${\bar{y}}_{IMU}$ are the mean angles of the shoulder calculated from each respective source.

#### 2.4.3. Skewness

The skewness of the velocity profile indicates whether the speed distribution is symmetric or biased in one direction. If the skewness is close to zero, the speed distribution is symmetric. If it is not zero, the distribution is biased in one direction. The skewness was calculated using the density function of the gamma distribution [23].

(3) $$\mu =\frac{\sum_{i=1}^{180}t_{i}\cdot v(t_{i})\cdot ∆t}{\sum_{i=1}^{180}v(t_{i})\cdot ∆t}.$$

(4) $$\sigma^{2}=\frac{\sum_{i=1}^{180}{\left(t_{i}-\mu \right)}^{2}\cdot v(t_{i})\cdot ∆t}{\sum_{n=1}^{180}v(t_{i})\cdot ∆t}.$$

(5) $$S=\frac{\sum_{i=1}^{180}{\left(t_{i}-\mu \right)}^{3}\cdot v(t_{i})\cdot ∆t}{\sigma^{3}\sum_{n=1}^{180}v(t_{i})\cdot ∆t}.$$

In this formula, μ is the expectation value of the velocity profile, $\sigma^{2}$ is the variance of the velocity profile, $v(t_{i})$ is the velocity profile, and ∆t is the measuring interval, i.e., 1/60 of a second. The skewness was calculated for the velocity profile within the 0–3 s interval, and the calculation was repeated every 6 s from the neutral position to the maximum elastic load.

### 2.5. Statistical Analysis

IBM SPSS Statistics 21 (IBM Corp., New York, NY, USA) was employed for data analysis using the two-way repeated analysis of variance (ANOVA) technique. In this analysis, the first factor was “hand”, representing the dominant hand (DH) and non-dominant hand (NDH), and the second factor was “intensity”, categorized into three levels: low (I1), moderate (I2), and high intensity (I3). This analysis determined the main effects of each factor and the interactions between the two factors. Statistical significance was set at <0.05. In cases where the main effects or interaction effects were statistically significant, post-hoc tests were conducted to determine where significant differences occurred. Bonferroni correction was applied to mitigate errors resulting from multiple comparisons. Additionally, the mean (M), standard error (SE), and standard deviation of the data were computed. Furthermore, Mauchly’s sphericity test assessed the assumption of sphericity for the ANOVA results obtained from repeated measurements.

## 3. Results

### 3.1. Comparison of Bilateral Performance in Lifting and Lowering Speeds at Various Intensities

**Figure 2:**
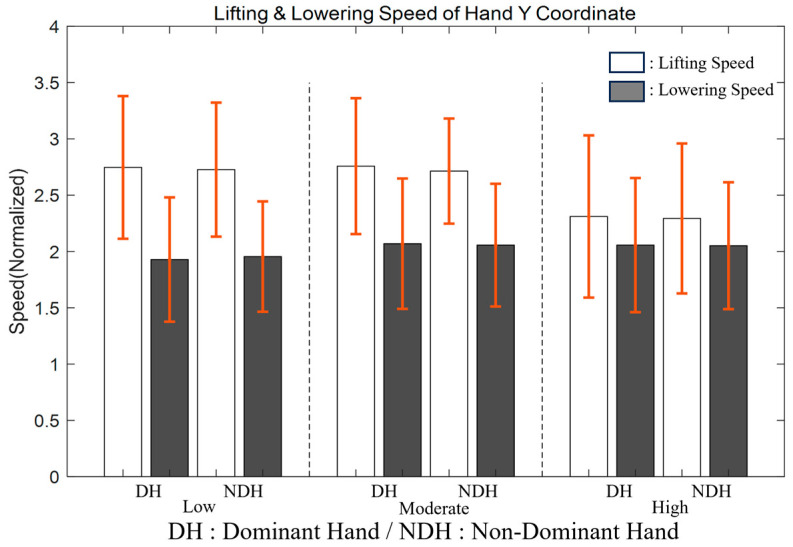
Boxplot for the lifting and lowering speeds at each intensity level and hand dominance.

In Section 3.1, our primary interest lies in assessing the symmetry between the DH and NDH. The study focused on lifting and lowering movements and aimed to investigate whether hand dominance would impact performance when participants interacted with a resistance of varying intensity levels. This inquiry was particularly relevant in proprioceptive feedback, where visual cues were absent, and the primary emphasis relied on the body’s internal sensory feedback mechanisms. Our analysis utilized a two-way repeated-measures ANOVA to discern any significant disparities in performance between DH and NDH across low, moderate, and high intensity levels (Figure 2). 

The boxplot comparison in Figure 2 illustrates normalized speeds for both lifting and lowering at each intensity level, categorized by hand dominance. The results of the two-way repeated-measures ANOVA indicated no significant interaction effect on the lifting speed of the hand’s y-coordinate (F(2,58) = 0.854, *p* = 0.431, partial $\eta^{2}$ = 0.029, as detailed in Supplementary Table S1). Specifically, no discernible performance differences were observed between DH and NDH across the intensity levels. At low intensity (I1), DH and NDH demonstrated equivalent lifting speeds (DH: M = 2.747, SE = 0.636; NDH: M = 2.727, SE = 0.595; *p* = 0.490), and this pattern persisted across moderate (I2) and high intensities (I3) with minimal variance. Likewise, the lowering speeds exhibited a similar trend, with no significant interaction effect between hand dominance and intensity (F(2,58) = 1.159, *p* = 0.321, partial $\eta^{2}$ = 0.038, as detailed in Supplementary Table S2). This finding indicates that the process of returning from the vertical target point to the starting position remained consistent between DH and NDH at all tested intensities. For instance, at low intensity, both hands displayed comparable results (DH: M = 1.928, SE = 0.552; NDH: M = 1.955, SE = 0.490; *p* = 0.205), a pattern that was also valid at moderate and high intensities. In summary, the lifting and lowering speeds between DH and NDH were statistically indistinguishable under various loading conditions. This suggests that hand dominance does not significantly impact the speed of arm movement coordination in tasks performed without visual feedback.

### 3.2. Comparison of Lifting Speed, Lowering Speed, and Range of Motion at Various Intensities

**Figure 3:**
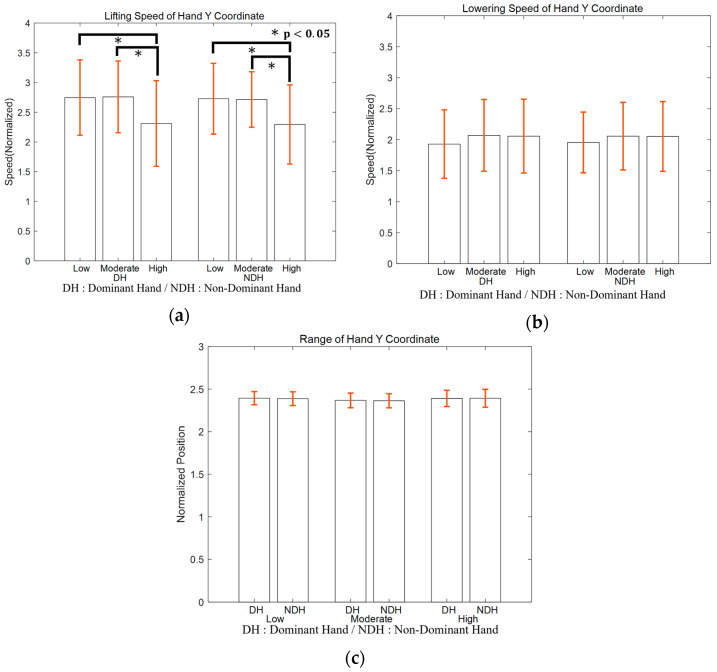
Boxplot for the (**a**) lifting speed, (**b**) lowering speed, and (**c**) range of motion at each intensity level.

This section expands beyond the bilateral symmetry focus discussed in Section 3.1. It delves into how the body reacts to differing levels of imposed load. By classifying intensity into three distinct levels—low, moderate, and high—we can pinpoint noteworthy variations in performance, especially in lifting speed, which exhibit significant differences at higher intensities. Figure 3 displays a boxplot analysis structured to elucidate the effects of exercise intensity on three kinematic parameters: lifting speed, lowering speed, and range of motion.

(a) Lifting Speed: The analysis revealed a noteworthy reduction in lifting speed at high intensity (I3), with the DH exhibiting a mean speed of 2.311 (SE = 0.720) and the NDH displaying a mean speed of 2.293 (SE = 0.666). This represents a significant decrease from the lifting speeds observed at low intensity (I1), where DH had a mean speed of 2.747 (SE = 0.636), and NDH had a mean speed of 2.727 (SE = 0.595). Both DH and NDH demonstrated statistically significant differences (DH: *p* = 0.001; NDH: *p* = 0.001) when comparing the high-intensity to low-intensity conditions.

A similar pattern of significant reduction in lifting speed was observed when comparing high intensity to moderate intensity (I2). DH had a mean speed of 2.758 (SE = 0.604), and NDH had a mean speed of 2.714 (SE = 0.467), with both DH and NDH again showing statistically significant differences (DH: *p* < 0.001; NDH: *p* < 0.001). However, it is worth noting that the transition from low to moderate intensity did not yield significant differences in lifting speed (DH: *p* = 1.00; NDH: *p* = 1.00). This suggests that the impact of exercise intensity on lifting speed becomes more pronounced at the higher end of the intensity spectrum.

(b) Lowering Speed: In contrast to the findings related to lifting speed, no significant differences were observed in the lowering speed across the various intensity levels. This consistent pattern was observed for both the DH, with a mean speed of 1.928 (SE = 0.552) at low intensity, and NDH, with a mean speed of 1.955 (SE = 0.490). These results suggest that the presence of elastic resistance does not significantly influence control during the lowering phase. Statistical analysis supported this observation, with no significant differences found for DH (*p* = 0.544 at moderate intensity and *p* = 0.303 at high intensity) or NDH (*p* = 0.066 at moderate intensity and *p* = 0.181 at high intensity) when comparing different intensity levels.

(c) Range of Motion: The range of motion remained consistent across all intensities, with low intensity (I1) demonstrating a mean value for DH at 2.394 (SE = 0.078) and for NDH at 2.388 (SE = 0.081). This consistency persisted through moderate intensity (I2) (DH: M = 2.367, SE = 0.086; NDH: M = 2.363, SE = 0.083) and high intensity (I3) (DH: M = 2.391, SE = 0.096; NDH: M = 2.393, SE = 0.105), with no statistically significant differences (DH: *p* = 0.150; NDH: *p* = 0.160 between low and moderate, and DH: *p* > 0.99; NDH: *p* > 0.99 between moderate and high).

In summary, these observations highlight that, except for a noticeable reduction in lifting speed at the highest intensity, the participants’ performance in terms of both speed and range of motion remained relatively unaffected by the intensity of the exercise.

### 3.3. Changes in the Velocity Profile with Varying Intensities

**Figure 4:**
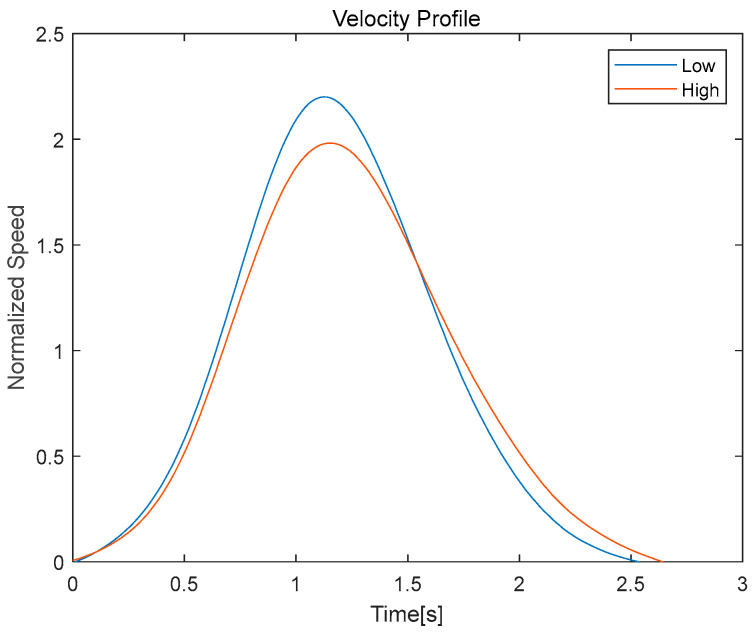
Modulation in velocity profiles with a shift in exercise intensity from a low to high level.

Among the kinematic data previously categorized into low, moderate, and high intensities, we specifically compare the velocity profiles under low- and high-intensity conditions. Figure 4 depicts the velocity profile of the lifting speed by intensity level.

In Table 2, indicators representing the characteristics of velocity profiles for each intensity level are organized and recorded.

The low-intensity condition exhibited a velocity profile with a skewness of 0.294, indicating a relatively symmetrical distribution of speeds with a peak velocity of 2.200 reached within 1.122 s after movement initiation. This profile was captured during the phase of motion, where participants stretched upwards in response to a metronome cue over a 3 s interval. Notably, the exercise duration for the low-intensity condition averaged 2.514 s.

By contrast, the high-intensity trials displayed a velocity profile with a skewness of 0.351, reflecting a slightly more asymmetrical distribution of speeds. The peak velocity observed was lower at 1.982, and the time to reach this peak was slightly longer, at 1.151 s. The exercise duration for these trials was, on average, marginally longer at 2.601 s. Comparing these two profiles underscores the impact of intensity on the dynamics of movement execution. Specifically, the increase in skewness and the decrease in peak velocity at higher intensities may suggest a shift in strategy to accommodate the greater resistance, potentially implicating the recruitment of different muscle groups or changes in neuromuscular control.

### 3.4. Comparative Analysis of Shoulder Angles Using OpenPose and IMU

**Figure 5:**
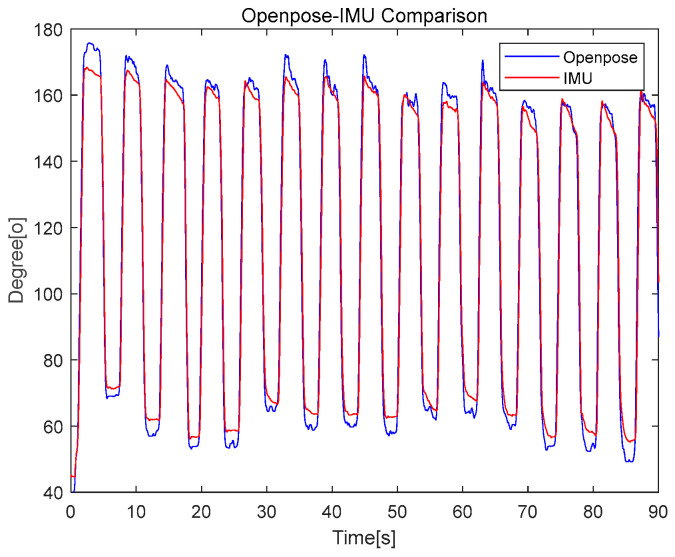
Alignment of shoulder angle measurements obtained from OpenPose and IMU during a series of upper arm movements.

To determine the positional accuracy of OpenPose in kinematic assessments, we conducted a comparison with the established IMU-based measurements of upper arm inclination angles during repetitive exercises. Figure 5 illustrates this comparison across 15 exercise repetitions, visually representing the agreement between the two methods.

In Table 3 we present a comparison of the results obtained from OpenPose and IMU, utilizing the root mean square error (RMSE) and the CC as key metrics for evaluation. Remarkably, the CC values, indicative of the degree of correlation between the two measurement techniques, consistently remained at a high level of 0.99 across all conditions. This remarkable level of correlation underscores an excellent agreement between the OpenPose and IMU methods, suggesting that OpenPose is a dependable tool for the real-time capture of upper arm kinematics.

The RMSE values, which ranged from 8.65 to 11.85, demonstrate a relatively narrow margin of error between the readings obtained from OpenPose and IMU across the participants. This consistent RMSE range indicates that OpenPose can estimate the upper arm shoulder angle with an accuracy similar to that of the IMU. Furthermore, the consistent maintenance of relatively low RMSE values strengthens the assertion that both methodologies closely align when estimating kinematic data.

## 4. Discussion

The findings of this study make a substantial contribution to our comprehension of motor control in situations lacking visual feedback, particularly in the presence of elastic loads. The notable preservation of symmetry in reaching movements, regardless of hand dominance, represents a remarkable aspect that calls into question the traditional understanding of proprioception’s role in motor coordination.

### 4.1. Symmetry under Elastic Load

Our experiment has substantiated that symmetry in reaching movements persists even when an elastic load is applied without visual information. Conventionally, bilateral coordination in movements with visual feedback has been acknowledged for its symmetry, attributed to the substantial influence of visual cues on trajectory generation and adjustment [24]. However, our study has unveiled that even without visual feedback, humans can uphold movement symmetry through proprioception. This finding suggests that internal motor control mechanisms are pivotal in preserving symmetry rather than relying solely on external visual feedback. The physical positioning and load detected under elastic resistance are communicated to both arms through commands generated in the primary motor cortex (M1). Arm dominance introduces disparities in the sensory information that each arm targets.

Previous research has indicated that the DH prioritizes visual targets, whereas the NDH prioritizes proprioceptive targets [25]. Consequently, in high-intensity exercises where muscle fatigue is a factor, it is anticipated that control by the NDH would become more prominent, potentially leading to asymmetric movements. However, our results demonstrate that even without visual targets, proprioception does not bestow dominance upon the NDH; instead, reaching control strives to engage both arms symmetrically, utilizing somatosensory feedback. This discovery, in contrast to prior theories, underscores the pivotal role of proprioception in upholding bilateral symmetry.

### 4.2. Elastic Load and Velocity Profile during Reaching

Our findings revealed that in the absence of visual feedback and when external forces were applied, the velocity profile during reaching movements exhibited a forward skew, indicative of an asymmetric form. This was observed through increased skewness and movement time, whereas the time required to reach peak velocity and the range of motion remained unchanged. These results imply that even without visual feedback, individuals adjust their speed to attain a position virtually generated by the brain’s internal model.

If we had observed that the velocity profile remained unaffected by external forces, as predicted by the minimum torque-change model, it could have suggested that humans prioritize muscle control generated by the brain’s internal model regardless of visual feedback. However, the results are per the minimum variance model, which posits that smaller target sizes result in speed control with an asymmetric velocity profile. This model is typically applied in situations involving visual feedback. However, our findings demonstrate its applicability even without visual feedback, underscoring the prioritization of reaching accuracy. Furthermore, as corroborated by our statistical analysis, the absence of significant differences in the movement range across different intensities suggests that the brain’s internal model prioritizes reaching accuracy over mitigating muscle fatigue induced by increased load.

### 4.3. Openpose Validation by Comparing with IMU

Two primary methodologies are commonly employed in biomechanical motion tracking: motion capture using OpenPose and the Inertial Measurement Unit (IMU) approach. In our study, the accuracy of OpenPose in estimating the upper arm shoulder angle was compared with that of the IMU. The Root Mean Square Error (RMSE) values, ranging from 8.65 to 11.85, demonstrate a relatively narrow margin of error between OpenPose and IMU measurements across participants. This consistency in RMSE values suggests that OpenPose can estimate the upper arm shoulder angle with an accuracy comparable to the IMU.

In the analysis of arm and forearm movements, OpenPose data exhibited fewer joint tracking errors under static conditions, while IMU data showed reduced errors during dynamic movements. Furthermore, the angles derived from video recordings using OpenPose displayed a robust positive correlation with those obtained through inertial sensors, with a correlation coefficient (CC) greater than 0.95. This strong correlation not only confirms the reliability of OpenPose in capturing accurate kinematic data but also strengthens the assertion that OpenPose offers valuable measurements for biomechanical motion tracking. These findings suggest that even with a single 2D camera, OpenPose provides reliable data that closely align with the measurements obtained from more complex IMU systems.

### 4.4. Limitations and Future Work

One of the primary limitations of our study was the overrepresentation of right-handed participants. This demographic bias could potentially limit the generalizability of our findings. Coordination dynamics vary between left-handed and right-handed individuals due to inherent neurological and biomechanical differences associated with handedness. Future research would benefit from a more balanced inclusion of left-handed individuals, which could offer a broader perspective on how handedness influences coordination in response to different force fields and proprioceptive demands.

Our study’s focus on proprioceptive feedback in movement coordination under elastic load aligns with the increasing emphasis on proprioception in rehabilitation. Research, such as that published Smith et al. [26], has underscored proprioceptive impairments in various populations and the potential benefits of proprioceptive training in enhancing motor function. Similarly, findings by Johnson and Lee [27] emphasize the significance of proprioception in neurological rehabilitation. These studies resonate with our observations, suggesting that exercises centered on proprioception, particularly in environments devoid of visual feedback, can play a crucial role in rehabilitation programs.

From a practical perspective, our findings indicate that rehabilitation exercises utilizing elastic resistance in a vertical plane can be effectively conducted with reduced reliance on visual feedback. This approach may be especially advantageous for patients recovering from injuries or conditions that impair their visual perception. By leveraging proprioceptive cues, therapists can tailor rehabilitation programs to enhance movement accuracy and control, thus compensating for reduced or absent visual inputs. This underscores the potential of proprioceptive training in improving motor control and facilitating recovery in visually impaired populations.

## 5. Conclusions

The findings of this study offer valuable insights into human motor control strategies during vertical plane elastic load reach movements, particularly when visual feedback is absent. The bilateral symmetry in performance across different intensities highlights the human capacity for precise proprioceptive-based movement control. Despite variations in the load intensity, participants consistently demonstrated symmetric reaching movements, indicating a robust internal model of movement execution within the brain.

However, an asymmetrical velocity profile, characterized by varying return durations at higher loads, suggests an adaptive strategy for controlled deceleration. This adaptation underscores the brain’s ability to adjust motor outputs in response to changing mechanical demands, ensuring accurate target achievement.

These results hold implications for the understanding of motor control in diverse sensory conditions and could inform rehabilitation practices where proprioception plays a critical role in the absence of visual cues.

## Figures and Tables

**Table 1:** Information on the participants.

	Male (n = 17)	Female (n = 13)	Total (n = 30)
Age (years)	24.0±4.2	26.2±4.9	24.9±4.6
Dominant hand	Right (n=17)	Right (n=12) Left (n=1)	Right (n=29) Left (n=1)
Body length (cm)	40.9±2.3	36.6±2.1	40.2±2.4
Left arm length (cm)	56.3±2.2	49.0±2.4	54.7±3.0
Right arm length (cm)	56.6±2.8	49.1±2.5	54.8±3.2
Maximal strength	18.3±3.19	8.1±2.5	13.9±5.8

**Table 2:** Skewness, peak velocity, and the time to peak velocity during reaching.

Intensity	Skewness	Peak Velocity	Time to Peak Velocity	Exercise Duration
Low	0.294	2.200	1.122	2.514
High	0.351	1.982	1.151	2.601

**Table 3:** Comparison Result of Openpose and IMU.

	1	2	3	4	5	6	7	8
RMSE	8.65	9.03	9.25	11.85	11.30	9.59	11.68	9.84
CC	0.99	0.99	0.99	0.99	0.99	0.99	0.99	0.99

## Data Availability

Data available on request due to privacy concerns. The data presented in this study are available on request from the corresponding author. The data are not publicly available because our experimental data consist of video recordings featuring participants’ faces, and several participants have refused to expose their faces.

## Supplementary Materials

The following supporting information can be downloaded at: https://www.mdpi.com/article/10.3390/bioengineering11010001/s1, Table S1: Summary of Statistical Analysis for Lifting Speed of the Hand Y Coordinate. Table S2: Summary of Statistical Analysis for Lowering Speed of the Hand Y Coordinate. Table S3: Summary of Statistical Analysis for Range of the Hand Y Coordinate.
