# Supplementary material for: Elastic Resistance and Shoulder Movement Patterns: An Analysis of Reaching Tasks Based on Proprioception
Source: Bioengineering (Basel). 2023 Dec 19;11(1):1. doi: 10.3390/bioengineering11010001 (PMC10813056; doi:10.3390/bioengineering11010001)
Supplement: Supplementary file 1 [file bioengineering-11-00001-s001.zip › bioengineering-2723310-supplementary.pdf]

Supplementary Table S1. Summary of the statistical analysis of Lifting Speed of the Hand y coordinate.

Item	Variable	Test	Statistic	Confidence
A	Lifting Speed under the conditions of each hand at each intensity	Two-way repeated measures ANOVA	Hand:<br>Mauchly's Test χ2(0) = 0.0, p = nothing, ε = 1;<br><br>F (1,29) = 0.797<br><br>Intensity:<br>Mauchly's Test χ2(2) = 14.187, p = 0.001, ε = 0.716;<br><br>F (1.431, 41.502) = 17.434;<br><br>Interaction:<br>Mauchly's Test χ2(2) = 1.574 p = 0.455, ε = 1;<br><br>F (2,58) = 0.854	Hand: p = 0.379, partial η2 = 0.027, power = 0.139, Sphericity assumed;<br><br>Intensity: p < 0.001, partial η2 = 0.375, power = 0.997, corrected by Greenhous-Geisser;<br><br>Interaction: p = 0.431, partial η2 = 0.029, power = 0.190, corrected by Huynh-Feldt;
B	Lifting Speed under the conditions of H1:H2 in I1	Bonferroni-corrected pairwise comparisons	H1: H2: t (29) = 0.699;	H1: H2; p = 0.490, Cohen’s d = 0.140;
C	Lifting Speed under the conditions of H1:H2 in I2	Bonferroni-corrected pairwise comparisons	H1: H2: t (29) = 1.163;	H1: H2; p = 0.254, Cohen’s d = 0.233;
D	Lifting Speed under the conditions of H1:H2 in I3	Bonferroni-corrected pairwise comparisons	H1: H2: t (29) = 0.341;	H1: H2; p = 0.736, Cohen’s d = 0.068;
D	Lifting Speed under the conditions of I1:I2, I1:I3, I2:I3 in H1	Bonferroni-corrected pairwise comparisons	I1: I2: t (29) = 0.267;<br>I1: I3: t (29) = 4.065;<br>I2: I3: t (29) = 5.294;	I1: I2; p = 1.000, Cohen’s d = 0.053;<br>I1: I3: p = 0.001, Cohen’s d = 0.813;<br>I2: I3: p < 0.001, Cohen’s d = 1.059;
E	Lifting Speed under the conditions of I1:I2, I1:I3, I2:I3 in H2	Bonferroni-corrected pairwise comparisons	I1: I2: t (29) = 0.330;<br>I1: I3: t (29) = 3.976;<br>I2: I3: t (29) = 5.164;	I1: I2; p = 1.000, Cohen’s d = 0.066;<br>I1: I3: p = 0.001, Cohen’s d = 0.795;<br>I2: I3: p < 0.001, Cohen’s d = 1.033;

Supplementary Table S2. Summary of the statistical analysis of Lowering Speed of the Hand y coordinate.

Item	Variable	Test	Statistic	Confidence
A	Lowering Speed under the conditions of each hand at each intensity	Two-way repeated measures ANOVA	Hand:<br>Mauchly's Test χ2(0) = 0.0, p = nothing, ε = 1;<br><br>F (1,29) = 0.101<br><br>Intensity:<br>Mauchly's Test χ2(2) = 0.811, p = 0.667, ε = 1;<br><br>F (2,58) = 2.706;<br><br>Interaction:<br>Mauchly's Test χ2(2) = 4.976 p = 0.083, ε = 1;<br><br>F (2,58) = 1.159	Hand: p = 0.753, partial η2 = 0.003, power = 0.061, Sphericity assumed;<br><br>Intensity: p = 0.075, partial η2 = 0.085, power = 0.515, Sphericity assumed;<br><br>Interaction: p = 0.321, partial η2 = 0.038, power = 0.245, Sphericity assumed;
B	Lowering Speed under the conditions of H1:H2 in I1	Bonferroni-corrected pairwise comparisons	H1: H2: t (29) = 1.296	H1: H2; p = 0.205, Cohen's d = 0.259
C	Lowering Speed under the conditions of H1:H2 in I2	Bonferroni-corrected pairwise comparisons	H1: H2: t (29) = 0.927	H1: H2; p = 0.362, Cohen's d = 0.185
D	Lowering Speed under the conditions of H1:H2 in I3	Bonferroni-corrected pairwise comparisons	H1: H2: t (29) = 0.150	H1: H2; p = 0.882, Cohen's d = 0.030
D	Lowering Speed under the conditions of I1:I2, I1:I3, I2:I3 in H1	Bonferroni-corrected pairwise comparisons	I1: I2: t (29) = 2.420<br>I1: I3: t (29) = 1.956<br>I2: I3: t (29) = 0.400	I1: I2; p = 0.066, Cohen's d = 0.484<br>I1: I3: p = 0.181, Cohen's d = 0.391<br>I2: I3: p = 1.000, Cohen's d = 0.080
E	Lowering Speed under the conditions of I1:I2, I1:I3, I2:I3 in H2	Bonferroni-corrected pairwise comparisons	I1: I2: t (29) = 1.369<br>I1: I3: t (29) = 1.695<br>I2: I3: t (29) = 0.098	I1: I2; p = 0.544, Cohen's d = 0.274<br>I1: I3: p = 0.303, Cohen's d = 0.339<br>I2: I3: p = 1.000, Cohen's d = 0.020

Supplementary Table S3. Summary of the statistical analysis of Range of the Hand y coordinate.

Item	Variable	Test	Statistic	Confidence
A	Range under the conditions of each hand at each intensity	Two-way repeated measures ANOVA	Hand:<br>Mauchly's Test $\chi2(0)$ = 0.0, p = nothing, ε = 1;<br><br>F (1,29) = 0.296<br><br>Intensity:<br>Mauchly's Test $\chi2(2)$ = 1.599, p = 0.450, ε = 1;<br><br>F (2,58) = 2.487<br><br>Interaction:<br>Mauchly's Test $\chi2(2)$ = 5.195, p = 0.074, ε = 1;<br><br>F (2,58) = 0.073	Hand: p = 0.591, partial η2 = 0.010, power = 0.082, Sphericity assumed;<br><br>Intensity: p = 0.092, partial η2 = 0.079, power = 0.480, Sphericity assumed;<br><br>Interaction: p = 0.930, partial η2 = 0.003, power = 0.061, Sphericity assumed;
B	Range under the conditions of H1:H2 in I1	Bonferroni-corrected pairwise comparisons	H1: H2: t (29) = 0.716;	H1: H2; p = 0.480, Cohen’s d = 0.143;
C	Range under the conditions of H1:H2 in I2	Bonferroni-corrected pairwise comparisons	H1: H2: t (29) = 0.459;	H1: H2; p = 0.649, Cohen’s d = 0.092;
D	Range under the conditions of H1:H2 in I3	Bonferroni-corrected pairwise comparisons	H1: H2: t (29) = 0.103;	H1: H2; p = 0.919, Cohen’s d = 0.021;
D	Range under the conditions of I1:I2, I1:I3, I2:I3 in H1	Bonferroni-corrected pairwise comparisons	I1: I2: t (29) = 2.014;<br>I1: I3: t (29) = 0.065;<br>I2: I3: t (29) = 1.995;	I1: I2; p = 0.160, Cohen’s d = 0.403;<br>I1: I3: p = 1.000, Cohen’s d = 0.013;<br>I2: I3: p = 0.167, Cohen’s d = 0.399;
E	Range under the conditions of I1:I2, I1:I3, I2:I3 in H2	Bonferroni-corrected pairwise comparisons	I1: I2: t (29) = 2.044;<br>I1: I3: t (29) = 0.162;<br>I2: I3: t (29) = 1.764;	I1: I2; p = 0.150, Cohen’s d = 0.409;<br>I1: I3: p = 1.000, Cohen’s d = 0.032;<br>I2: I3: p = 0.265, Cohen’s d = 0.353;
